# Characteristics of Milk Fermented by *Streptococcus thermophilus* MGA45-4 and the Profiles of Associated Volatile Compounds during Fermentation and Storage

**DOI:** 10.3390/molecules23040878

**Published:** 2018-04-11

**Authors:** Tong Dan, Rulin Jin, Weiyi Ren, Ting Li, Haiyan Chen, Tiansong Sun

**Affiliations:** Key Laboratory of Dairy Biotechnology and Engineering, Ministry of Education, Inner Mongolia Agricultural University, 306 Zhaowuda Street, Hohhot 010018, China; dantong813218@imau.edu.cn (T.D.); jrl_happy@163.com (R.J.); renweiyi153@163.com (W.R.); 18848114353@163.com (T.L.); m15024943239@163.com (H.C.)

**Keywords:** *Streptococcus thermophilus*, fermented milk, fermentation characteristics, solid-phase microextraction (SPME), gas chromatography/mass spectrometry (GC-MS)

## Abstract

The lactic acid bacterium *Streptococcus thermophilus* is a major starter culture for the production of dairy products. In this study, the physiochemical characteristics of milk fermented by the MGA45-4 isolate of *S. thermophilus* were analyzed. Our data indicate that milk fermented using *S. thermophilus* MGA45-4 maintained a high viable cell count (8.86 log10 colony-forming units/mL), and a relatively high pH (4.4), viscosity (834.33 mPa·s), and water holding capacity (40.85%) during 14 days of storage. By analyzing the volatile compound profile using solid-phase microextraction and gas chromatography/mass spectrometry, we identified 73 volatile compounds in the fermented milk product, including five carboxylic acids, 21 aldehydes, 13 ketones, 16 alcohols, five esters, and 13 aromatic carbohydrates. According to the odor activity values, 11 of these volatile compounds were found to play a key role in producing the characteristic flavor of fermented milk, particularly octanal, nonanal, hexanal, 2,3-butanedione, and 1-octen-3-ol, which had the highest odor activity values among all compounds analyzed. These findings thus provide more insights in the chemical/molecular characteristics of milk fermented using *S. thermophilus*, which may provide a basis for improving dairy product flavor/odor during the process of fermentation and storage.

## 1. Introduction

*Streptococcus thermophilus* is a Gram-positive, non-pathogenic, facultative anaerobic lactic acid bacterium (LAB). This bacterium is related to other LABs, such as *Lactococcus lactis*, which is the most important industrial starter culture widely used in the dairy industry [1]. Among the opportunistic bacterial pathogens that are used for the preparation of dairy products, *S*. *thermophilus* is the only species generally recognized as being safe [2]. This species can be used alone or in combination with other LABs for the production of dairy products such as cheese [3,4], yogurt [5] and fermented camel milk [6]. Therefore, *S*. *thermophilus* has been proven to have industrial value [2].

*Streptococcus thermophilus* has the ability to metabolize lactose into exopolysaccharides, vitamins, and several flavor compounds [7]. In dairy products, multiple categories of volatile organic aroma compounds have been identified including carboxylic acids, aldehydes, ketones, alcohols, and esters [8,9,10,11]. These volatile compounds provide the texture, mouthfeel, and taste/odor of dairy products and therefore they are the key determining factors of product quality [12,13,14,15]. Although many volatile compounds have been identified, only a few, such as acetic acid, are considered to have a significant influence on the final flavor of dairy products. Acetic acid can be produced during storage at low temperatures and it is the major component responsible for increasing the acidic flavor of many dairy products [16]. Recent studies have demonstrated that acetic acid is frequently found in fermented milk [16], fermented camel milk [6], and in goat cheese [8].

To better elucidate how much each volatile compound contributes to the overall flavor of a sample, the odor activity value (OAV) has been developed and applied to the product evaluation process [17,18]. OAVs are represented as the ratios between the concentration of each compound and its detection threshold concentration [19]. If the OAV of a flavor compound is greater than 1, the compound is defined as an active odorant. In a recent study, several compounds with an OAV greater than one were identified, which may have an important impact on the aroma of fermented cow milk [20].

The main advantages of solid-phase microextraction (SPME) as a pretreatment method are its simplicity, low cost, ease of automation, and solvent-free extraction [16]. SPME combined with gas chromatography–mass spectrometry (SPME-GC-MS) has been used to study the volatile composition of a wide range of products [20].

Industrial strains of LAB can be isolated from different milk environments, including fermented milk, cheese, and kefir [21]. In China, yogurt has become a major fermented dairy product. However, few studies have examined the profile of volatile compounds in milk fermented with LAB strains isolated from traditional dairy products. Therefore, this study investigated aroma formation and the fermentation characteristics of milk that had been fermented by *S*. *thermophilus* MGA45-4 from traditional fermented milk collected in Kent Province, Mongolia and tested its behavior as a starter in the yogurt industry [22].

## 2. Results and Discussion

### 2.1. Physicochemical Characteristics of Milk Fermented Using S. thermophilus 

**Figure 1:**
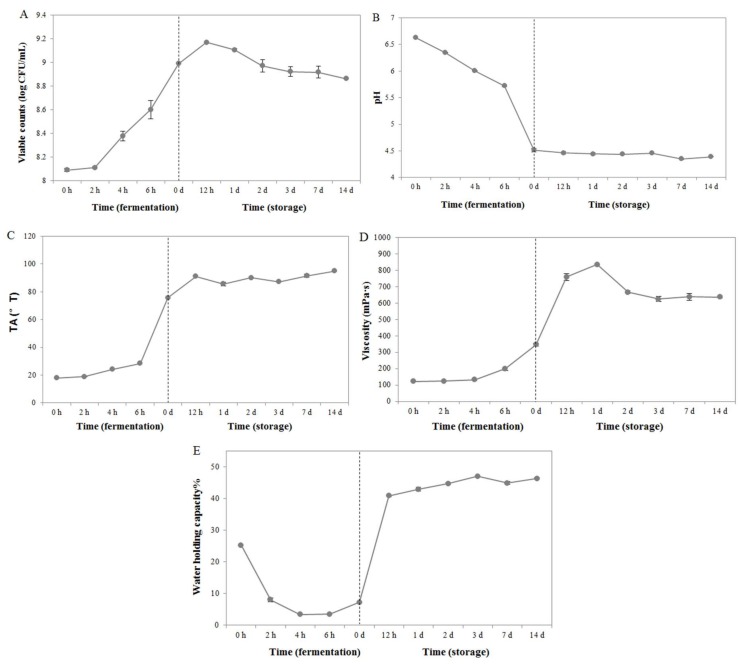
The physiochemical characteristics of milk fermented by *S. thermophilus* MGA45-4 during fermentation (0 h, 2 h, 4 h, and 6 h) and storage (0 d, 12 h, 1 d, 2 d, 3 d, 7 d, and 14 d). The parameters analyzed included (**A**) the count of viable *S. thermophilus* MGA45-4 cells, (**B**) pH, (**C**) titratable acidity, (**D**) viscosity, and (**E**) water-holding capacity of the fermented milk samples.

To evaluate the fermentation characteristics of *S*. *thermophilus* MGA45-4, we measured the viable cell counts, pH, TA, WHC, and viscosity of fermented milk samples. The trends for changes in viable cell counts differed between fermentation and storage (Figure 1A). Specifically, while the viable cell count increased gradually during fermentation, it peaked at the 12th day during storage (9.1688 log_10_ CFU/mL), followed by a steady decline (Figure 1A). The pH of samples decreased from 6.63 to 4.5 within the first 6.5 h of fermentation (Figure 1B). This value remained almost unchanged (4.4) during the 14 days of storage, suggesting that carboxylic acid production by *S*. *thermophilus* MGA45-4 is inhibited at a refrigeration temperature of 4 °C. Unlike pH, the TA value increased steadily during fermentation, reaching 75.72 °T at the final stage of the process (Figure 1C). A continuous but smaller increase was observed in TA during storage, with the value changing from 75.72 °T on the first day to 95.07 °T on the 14th day of storage (Figure 1C). During the fermentation period, the viscosity of fermented milk increased significantly over time and a maximum value of 834.33 mPa·s was reached on day 1 of storage (Figure 1D). Overall, the fermented milk had a significantly higher viscosity in storage than during fermentation. Consistent with this, milk samples in storage also had a markedly higher water-holding capacity (WHC) compared with those in the fermentation process (Figure 1E). On the third day of storage, the WHC reached a maximum value of 48.01%, which was significantly greater than the WHC obtained from the other time points (Figure 1E). Sensory assessment indicated that products stored for 0 d and 12 h at 4 °C were better than the other samples.

### 2.2. Analysis of Volatile Compounds in Fermented Milk 

Odor/flavor formation in dairy products is a complex process which involves glycolysis, lipolysis, and proteolysis of various chemical components. Depending on the starter culture isolates used for fermentation, the final quality of fermented milk can vary significantly. To evaluate the aroma profile of milk fermented by *S. thermophilus* MGA45-4, we used the SPME-GC/MS technique to analyze the volatile compound composition of samples during fermentation and storage. As shown in Table 1, we identified 73 compounds based on the retention indexes (RIs) calculated using the HP-5MS column. These compounds included various types of carboxylic acids, aldehydes, ketones, alcohols, esters, and aromatic carbohydrates. The range of the RI for almost all compounds was ±5 or ±10 units.

Acids are the precursors of methyl ketones, alcohols, lactones, and esters; as such, they are important for the generation of odors in dairy products [23]. Using the SPME pretreatment method combined with GC/MS, we identified five different carboxylic acids from the *S. thermophilus* MGA45-4 fermented milk (Table 1). During the storage period, two short-chain fatty acids, acetic acid and hexanoic acid, were detected, which ranged in concentration from 17.21–70.07 μg/L, and 7.36–35.17 μg/L, respectively. Given their strong odor, short-chain fatty acids (C < 6) are particularly crucial for odor generation in fermented milk compared with other acid compounds [24]. Previous studies have demonstrated that acetic acid is responsible for the vinegary, pungent, acidic odor associated with dairy products [25], while hexanoic acid has a sickening, sweet, rancid, cheese-like odor [26]. Two additional acids, nonanoic and octanoic acids, were found in samples from the storage process, but were absent from those undergoing fermentation (Table 1). In particular, the concentration of octanoic acid reached as high as 3.64 μg/L on the second day of storage, which was consistent with earlier findings reported by Condurso et al. [8].

Based on the SPME/GC/MS analysis, we identified 21 types of aldehydes in the volatile compound profile (Table 1). Aldehyde compounds are short-lived constituents of dairy products as they are rapidly converted into the corresponding alcohols or acids such as 3-methylbutanal and hexanal upon production [27,28]. As shown in Table 1, high levels of 3-methyl-butanal (7.52–16.95 µg/L) were detected in milk samples during fermentation. This is in agreement with the earlier finding that 3-methylbutanal is an important determinant of odor in dairy products [29]. We also detected a significant amount of benzaldehyde (5.65–7.14 μg/L) in samples undergoing fermentation. Benzaldehyde is produced from phenylacetaldehyde through α-oxidation or from cinnamic acid through β-oxidation [30]. This compound is also frequently found in dairy products such as cheese [8], fermented milk [18], and fermented camel milk [6].

The predominant group of volatile compounds found in dairy products is ketones, such as 2,3-butanedione which can be produced from methyl-ketones through α-oxidation of fatty acids [31]. In this study, we identified 13 ketone compounds in samples from the fermentation and storage stages. Based on their concentrations, the main ketone compounds included 2,3-butanedione (9.03–15.25 μg/L), acetoin (3.22–58.48 μg/L), 2-heptanone (17.43–53.88 μg/L), and 2-nonanone (5.95–32.78 μg/L). 2,3-Butanedione and acetoin were present in both the fermentation and storage stages, except at the end of storage. Acetoin can be produced from 2,3-butanedione as a byproduct of LAB metabolism, and both of these compounds can be further converted into other metabolites such as 2-butanone, 2-butanol, and butane-2,3-diol [32]. Our data were similar to those of an earlier report which indicated that 2,3-butanedione and acetoin can be detected in fermented milk and may be important for the final determination of product odor [11].

In addition to ketones, the type and amount of alcohol compounds produced can also have a significant impact on the aroma of dairy products. Some alcohol compounds can be derived from amino acids or aldehydes. For example, 3-methyl-butanol is generated through the reduction of the corresponding aldehydes [33]. In our study, 17 types of alcohol compounds were identified in the aroma profile of fermented milk (Table 1). Based on their concentrations, the main alcohol compounds were 3-methyl-butanol (4.28–23.37 μg/L), hexanol (38.35–152.25 μg/L), heptanol (58.68–244.29 μg/L), and 1-nonanol (12.11–45.23 μg/L). 1-Octen-3-ol (1.09–9.88 μg/L) was detected in samples undergoing fermentation and during storage. This compound produces a powerful, sweet, and earthy odor and, therefore, it may play a key role in generating the characteristic flavor of fermented milk [34].

Ester compounds are another group of volatile constituents that influence the odor outcome of dairy products [35]. The substrates required for the biosynthesis of ester compounds are mostly generated through the metabolism of carbohydrates and fat as well as catabolism of amino acids [36]. In the volatile fraction of samples analyzed, we identified five ester compounds and the main ester types included acetic acid octyl ester (1.23–7.83 μg/L), formic acid octyl ester (32.29–132.2 μg/L), and formic acid hexyl ester (1.01–2.66 μg/L) (Table 1). Esters contribute to the generation of fruity odors such as apple-like, pear-like, and banana-like odors [37]. Due to their relatively low detection threshold values (e.g., the threshold value of acetic acid octyl ester is 12 μg/L) [38], they can have a significant impact on the aroma of dairy products.

Aromatic carbohydrates can be characterized directly by GC-MS because the degradation process of these compounds is relatively slow [39]. Using GC-MS, we identified 13 aromatic hydrocarbon compounds in milk fermented by *S*. *thermophilus* MGA45-4. Those with a relatively high concentration were heptane (2.21–8.38 μg/L), 1-octene (3.94–5.91 μg/L), and 1-nonene (5.48–46.1 μg/L), which were consistent with previously reported findings [16].

### 2.3. Evaluation of OAVs

To determine how each volatile compound contributed to the overall odor profile of milk fermented by *S*. *thermophilus* MGA45-4, we calculated the OAVs for all samples during fermentation and storage (Table 2). Compounds with an OAV greater than one are considered to have a greater influence on the product’s odor and flavor [20]. Among the 73 volatile compounds identified, some compounds were found to have a higher concentration than their detection threshold concentration (i.e., OAV > 1). Specifically, seven aldehyde compounds exhibited an OAV > 1, including octanal (4.21–127.93), nonanal (1.03–52.04), hexanal (1.32–33.03), (E)-2-octenal (1.16–6.83), 3-methyl-butanal (1.39–2.34), (E)-2-heptenal (0.23–1.81), and (E)-2-pentenal (0.85–1.61). This indicated that aldehydes are important contributors to the flavor of milk fermented by *S. thermophilus* MGA45-4. Of the 16 ketone compounds identified, only 2,3-butanedione (0.90–1.65) and acetoin (0.06–1.06) had an OAV > 1. Given that 2,3-butanedione is a well-known metabolite of LAB, it is likely that it makes a significant contribution to the good odor/flavor of milk fermented by *S*. *thermophilus* MGA45-4 [40]. Although alcohol compounds represent the largest group of volatile compounds identified in our study, only hexanol and 1-octen-3-ol had an OAV greater than one, suggesting that they may play a role in the odor/flavor of related dairy products [6]. This result is consistent with Attaie [41].

Almost all of the acid compounds that had a higher reported threshold value had low concentrations in the milk fermented by *S*. *thermophilus* MGA45-4. For example, the threshold value of acetic acid was 32,300 μg/L and the OAV was <1 (0.00053–0.00217). Similar acid compounds were hexanoic acid (threshold value, 27,100 μg/L) and octanoic acid (threshold value, 11,300 μg/L) [42].

The physicochemical characteristics of fermented milk can affect the release of volatile compounds from the microstructure of food matrices. Most of the key flavor compounds (OAV >1) were detected on day 0 of storage. This result also indicated that the flavor on day 0 of storage was better than at other time points. On day 0 of storage, although the pH of the samples decreased to 4.5, the TA increased to 75.72 °T, and the viable cell count increased to 8.99 log_10_ CFU/mL. These changes in the physicochemical characteristics enable the release of flavored compounds. The results of sensory assessment are consistent with this conclusion.

## 3. Experimental

### 3.1. Bacterial Isolates and Reagents

*Streptococcus thermophilus* MGA45-4 was originally isolated from traditional fermented milk collected in Kent Province, Mongolia, and was used throughout this study [22]. Standard *n*-alkanes (C_3_–C_25_) were obtained from AccuStandard (New Haven, CT, USA). 1,2-Dichloro-benzene was used as an internal standard (ISTD) and was purchased from Sigma–Aldrich (Steinheim, Germany). MRS (De Man, Rogosa and Sharpe) broth was purchased from OXOID (Hampshire, England) and whole milk powder was purchased from NZMP (Wellington, New Zealand).

### 3.2. Preparation of Fermented Milk

Fermented milk was prepared using a previously described method [40] with some modifications. Briefly, frozen *S*. *thermophilus* MGA45-4 cells were propagated routinely by culturing in MRS broth for 24 h at 37 °C at least three times, followed by inoculation in milk/sucrose medium. Sterile milk was prepared by reconstituting 11.5% (*w/v*) whole milk powder in distilled water and heating to 50 °C for approximately 30 min, followed by supplementation with 6.5 g/100 g of sucrose. The resultant medium was sterilized by heating at 95 °C for 5 min and stored at 4 °C before use. *S*. *thermophilus* MGA45-4 was inoculated at a concentration of 5 × 10^7^ colony-forming units (CFU)/mL. After the milk coagulated, samples were incubated at 42 °C until the pH dropped to 4.5 and were then stored at 4 °C for 14 days. Samples were taken from each culture after 0 h, 2 h, 4 h, and 6 h of fermentation and 0 d, 12 h, 1 d, 2 d, 3 d, 7 d, and 14 d of storage. The fermented milk samples were frozen at −20 °C before analysis. 

### 3.3. Determining Viable Cell Counts 

Viable *S. thermophilus* MGA45-4 cells were counted using the pour-plate method, as previously described [46]. To enumerate viable cells, a 1 mL sample was diluted in 9 mL of sterile physiological saline (0.85%, *w/v*) and suitable dilutions were plated on MRS agar. Plates were incubated under anaerobic conditions at 42 °C for 48 h. Colonies were counted on each plate and viable cell counts were expressed as log_10_ CFU/mL.

### 3.4. Determination of pH and TA

The pH was measured at 20 °C using a pHS-3C precision pH meter (Leici Devices, Shanghai, China). TA was determined as previously described [47]. Five-gram samples were weighed and mixed with 40 mL of water and then titrated with standardized 0.1 N NaOH to a pH of 8.1 using 0.5% phenolphthalein as an indicator.

### 3.5. Determination of Viscosity

Viscosity was measured using a Brookfield DV-E Viscometer (Brookfield Engineering Laboratories, Middleboro, MA, USA). All samples were spun at 100 rpm for 30 s using a Brookfield DV-1 viscometer with a No. 4 spindle [48]. The viscosity was expressed in mPa·s.

### 3.6. Determination of WHC

The WHC is defined as the ability of fermented milk to hold all or part of its own water [49]. Fermented milk (20 g) was placed in a funnel containing filter paper and allowed to run through at 21 °C for 120 min. The filtrate was collected and weighed. The WHC was calculated using the following equation:

WHC (%) = (1 − W1/W2) × 100%,

where W1 = weight of filtrate in grams; and W2 = weight of fermented milk in grams.

### 3.7. HS-SPME-GC-MS Analysis

An SPME fiber (50/30 μm divinylbenzene/Carboxen/polydimethylsiloxane; Supelco, Bellefonte, PA, USA) was tested and used to identify the volatile compounds produced by *S*. *thermophilus* MGA45-4 in the fermented milk samples according to described methods [16]. The fiber was exposed for 60 min in the headspace of 20 mL glass vials (CNW Technologies, Germany) fitted with a polytetrafluoroethylene/silicone septum. Each glass vial contained 5 mL of sample and 10 μg/L of the ISTD. Subsequently, the fiber was immediately placed in the injection port of a 7890B GC system (Agilent Technologies, Palo Alto, CA, USA) for 5 min at 270 °C to allow for desorption of the volatile compounds into the GC.

### 3.8. Identification of Volatile Compounds

Absorbed volatiles were analyzed using a 7890 B GC equipped with a 5977 A mass selective detector (MSD; Agilent Technologies) and an HP-5MS column (30-m length, 0.25-mm i.d., 0.25-μm film thickness; Agilent Technologies). Helium was used as the carrier gas at 1 mL/min. The oven temperature was set at 35 °C for 5 min, increased to 140 °C at a rate of 4 °C/min for 5 min, and then increased further to 250 °C at a rate of 10 °C/min. A final 5-min extension was performed at 250 °C. The ion source and the transfer line temperatures were set at 230 °C and 250 °C, respectively. The mass spectra of the samples were recorded with a scan range of 40–400 m/z and electronic impact (EI) mode at 70 eV. *n*-Alkanes (C3–C25) were used under the same experimental conditions to calculate the RIs of the volatile aroma compounds in each sample. All volatile compounds were semi-quantified and the results are shown as the retention time and relative peak area.

### 3.9. Determination of OAV

The OAV refers to the ratio of the concentration of a compound to its detection threshold concentration [19]. The OAVs in this study were calculated as previously described [20].

### 3.10. Sensory Evaluation

The flavors of the fermented milk products were assessed by six trained panelists. A beaker (100 mL) filled with the sample was used for evaluation. Flavor intensity was recorded on a scale ranging from 1 (strongly attractive) to 5 (strongly unappealing), according to international standards. Water was provided for mouth washing between samples.

### 3.11. Statistical Analysis

The data were analyzed with ANOVA using Proc Mixed (SAS Institute, Cary NC, USA). Significant differences between means were determined using Fisher’s protected least significant difference test. Significant differences were defined at *p* < 0.05 (SAS Institute, 1998). All measurements were performed in triplicate.

## 4. Conclusions

In this study, we characterized the physiochemical properties of milk fermented by *S*. *thermophilus* MGA45-4 by monitoring the viable cell count, pH, TA, viscosity, and WHC of milk samples during fermentation and storage at 4 °C. Our data indicate that the product had a viable count of >8.86 log_10_ CFU/mL during both the fermentation and storage stages. A pH of 4.4 and viscosity of 834.33 mPa·s were maintained in the storage stage. By analyzing the volatile compound profile of fermented milk using SPME-GC-MS, we identified 73 volatile compounds including 5 carboxylic acids, 21 aldehydes, 13 ketones, 16 alcohols, 5 esters, and 13 aromatic carbohydrates from samples undergoing fermentation and during storage. As indicated by their OAVs, some compounds were found to be the key factors determining the product odor/flavor. In particular, octanal, nonanal, hexanal, 2,3-butanedione, and 1-octen-3-ol, which had the highest OAVs among all compounds analyzed, likely contributed the most to the characteristic flavor of fermented milk. Together, our study provides an additional chemical/molecular basis for a better understanding of the aroma characteristics of fermented milk. This in turn may help improve the flavor quality of dairy products during the process of fermentation and storage.

## Figures and Tables

**Table 1:** Volatile compounds produced in milk fermented by *S. thermophilus* MGA45-4 during fermentation and storage.

	Volatile Compound	Chemical Formula	RT ^1^	RI ^2^	RI ^3^	Method ^4^	μg/L										
							0 h (F) ^5^	2 h (F)	4 h (F)	6 h (F)	0 d (S) ^6^	12 h (S)	1 d (S)	2 d (S)	3 d (S)	7 d (S)	14 d (S)
**Carboxylic acid compounds**																	
1	Acetic acid	C_2_H_4_O_2_	2.71	630.71	638	MS, RI	-	-	-	-	70.07	24.76	21.08	24.28	19.80	18.19	17.21
2	Hexanoic acid	C_6_H_12_O_2_	14.72	1021.01	1020	MS, RI	-	-	-	-	35.17	29.77	7.68	7.49	7.89	7.52	7.36
3	Heptanoic acid	C_7_H_14_O_2_	15.80	1053.46	1062	MS, RI	-	-	-	-	8.31	4.34	3.05	3.47	3.33	2.15	3.20
4	Nonanoic acid	C_9_H_18_O_2_	21.09	1224.43	1226	MS, RI	-	-	-	-	-	-	-	0.75	0.18	-	-
5	Octanoic acid	C_8_H_16_O_2_	20.21	1193.85	1191	MS, RI	-	-	-	-	-	-	-	3.64	-	-	-
**Aldehydes**																	
1	Methacrolein	C_4_H_6_O	2.30	599.69	567	MS, RI	-	-	-	1.38	-	-	-	-	-	-	-
2	Butanal, 3-methyl-	C_5_H_10_O	3.47	689.08	689	MS, RI	7.52	15.02	16.95	12.62	-	-	-	-	-	-	-
3	Butanal, 3-hydroxy-	C_4_H_8_O_2_	3.53	693.79	-	MS	-	-	-	-	4.45	6.83	3.59	1.79	-	-	-
4	2-Pentenal, (E)-	C_5_H_8_O	5.52	765.69	765	MS, RI	-	-	-	1.93	1.15	1.03	1.02	1.23	1.25	1.60	1.88
5	Hexanal	C_6_H_12_O	6.87	809.39	809	MS, RI	99.08	91.58	58.12	52.03	3.96	-	-	-	-	-	-
6	2-Pentenal, 2-methyl-	C_6_H_10_O	7.53	826.57	826	MS, RI	-	-	-	-	1.89	-	-	-	-	-	-
7	2-Hexenal, (E)-	C_6_H_10_O	8.95	863.25	861	MS, RI	1.91	1.92	1.96	5.58	6.33	2.82	1.89	2.03	1.71	1.71	1.93
8	Heptanal	C_7_H_14_O	10.87	905.14	906	MS, RI	191.95	129.22	78.60	32.93	4.41	1.84	1.01	1.25	1.73	1.34	2.25
9	2-Heptenal, (E)-	C_7_H_12_O	12.87	965.33	964	MS, RI	10.03	11.41	10.42	11.53	23.47	7.45	3.39	6.20	2.97	4.90	5.78
10	Benzaldehyde	C_7_H_6_O	12.94	970.87	971	MS, RI	7.14	6.81	5.65	-	-	-	-	-	-	-	-
11	Octanal	C_8_H_16_O	14.57	1009.21	1005	MS, RI	89.55	45.12	23.49	18.24	7.73	2.95	-	-	-	-	-
12	2,4-Heptadienal, (E,E)-	C_7_H_10_O	14.81	1023.54	1023	MS, RI	-	1.42	1.85	1.40	1.15	-	-	-	-	-	-
13	2-Octenal, (E)-	C_8_H_14_O	16.41	1069.45	1067	MS, RI	13.29	13.86	15.07	15.66	20.48	10.21	4.74	4.44	3.58	3.49	4.28
14	Nonanal	C_9_H_18_O	17.92	1118.91	1119	MS, RI	52.04	35.95	28.60	9.58	4.01	1.52	1.43	1.03	1.50	1.12	1.13
15	2-Nonenal, (E)-	C_9_H_16_O	19.62	1172.50	1172	MS, RI	26.20	30.44	22.48	25.42	31.14	18.42	6.26	12.15	4.62	6.46	6.58
16	Decanal	C_10_H_20_O	21.02	1216.62	1214	MS, RI	3.63	2.83	2.59	1.87	-	-	-	-	-	-	-
17	2-Decenal, (Z)-	C_10_H_18_O	22.61	1277.90	1280	MS, RI	12.13	15.01	19.53	22.75	28.22	16.54	7.93	7.64	7.42	6.07	7.02
18	2-Decenal, (E)-	C_10_H_18_O	22.75	1281.57	1279	MS, RI	3.80	6.69	19.01	-	-	-	-	-	-	-	-
19	Undecanal	C_11_H_22_O	23.51	1310.47	1300	MS, RI	-	-	-	-	1.94	-	-	-	-	-	-
20	2-Undecenal, E-	C_11_H_20_O	24.55	1349.47	1340.00	MS, RI	-	-	-	1.56	-	-	-	-	-	-	-
21	2-Undecenal	C_11_H_20_O	24.81	1359.26	1359	MS, RI	2.12	3.50	6.48	6.25	8.91	4.28	3.65	3.53	2.44	2.02	2.22
**Ketones**																	
1	2,3-Butanedione	C_4_H_6_O_2_	2.16	STD	MS. STD	MS, RI	-	-	-	-	16.08	16.49	15.25	16.07	9.03	-	-
2	2-Butanone, 3-methyl-	C_5_H_10_O	2.26	STD	MS. STD	MS, RI	-	3.34	5.89	18.97	16.91	16.13	10.85	10.25	8.54	7.84	7.01
3	2-Pentanone	C_5_H_10_O	3.42	685.33	687	MS, RI	-	-	-	-	-	-	-	0.78	-	-	-
4	Acetoin	C_4_H_8_O_2_	4.22	720.87	MS. STD	MS	-	-	3.22	20.24	58.48	27.30	29.87	24.10	21.12	24.38	23.98
5	3-Pentanone, 2-methyl-	C_6_H_12_O	5.23	755.70	752	MS, RI	-	-	-	-	-	-	-	1.88	-	-	-
6	3-Hexen-2-one	C_6_H_10_O	8.51	852.38	845	MS, RI	-	-	-	-	3.07	1.40	1.54	1.49	0.80	0.98	1.19
7	2-Hexanone, 5-methyl-	C_7_H_14_O	8.97	864.56	857	MS, RI	-	-	-	14.49	-	-	-	-	-	-	-
8	2-Heptanone	C_7_H_14_O	10.43	891.82	891	MS, RI	31.60	32.13	32.41	31.62	53.88	26.19	19.54	17.78	18.53	17.43	17.66
9	3-Heptanone, 5-methyl-	C_8_H_16_O	12.87	968.76	962	MS, RI	2.60	2.79	3.12	3.09	-	-	-	-	-	-	-
10	2-Octanone	C_8_H_16_O	14.15	1003.70	1003	MS, RI	14.44	15.14	15.44	15.34	17.96	9.16	7.58	7.34	7.55	7.19	7.22
11	3-Octen-2-one	C_8_H_14_O	15.79	1049.21	1046	MS, RI	7.55	7.83	7.85	7.52	9.88	5.00	4.98	4.53	1.18	-	-
12	2-Nonanone	C_9_H_18_O	17.54	1098.98	1096	MS, RI	26.03	28.38	32.78	28.02	26.66	13.78	7.39	5.95	-	-	-
13	2-Undecanone	C_11_H_22_O	23.52	1310.70	1305	MS, RI	3.41	3.48	3.89	3.33	3.90	2.18	1.78	1.45	1.70	1.10	1.43
**Alcohols**																	
1	Butanol, 3-methyl-	C_5_H_12_O	5.71	772.37	767	MS, RI	5.17	4.28	5.03	18.39	23.37	10.80	10.76	10.83	10.39	11.29	11.09
2	1-Pentanol	C_5_H_12_O	5.71	772.37	772	MS, RI	-	-	-	-	-	-	-	-	-	-	10.95
3	2-Heptanol, 3-methyl-	C_8_H_18_O	7.23	818.65	-	MS	-	-	-	-	2.88	2.56	2.69	2.06	-	-	-
4	Hexanol	C_6_H_14_O	9.66	882.39	880	MS, RI	40.75	42.70	49.69	53.72	152.25	74.39	44.01	38.35	43.49	43.49	41.13
5	Heptanol	C_7_H_16_O	13.26	979.30	974	MS, RI	58.68	121.58	142.23	196.00	244.29	121.75	82.17	71.20	70.59	72.54	68.06
6	cis-Hept-4-enol	C_7_H_14_O	13.26	979.36	-	MS	-	2.54	2.21	3.79	7.84	4.11	1.75	1.47	1.47	1.52	0.96
7	1-Octen-3-ol	C_8_H_16_O	13.75	991.78	986	MS, RI	4.93	7.35	4.47	5.78	9.88	4.16	2.21	1.66	1.10	1.87	1.09
8	3,5-Octadien-2-ol	C_8_H_14_O	15.32	1039.01	1037	MS, RI	-	-	-	-	10.36	6.66	1.67	1.78	1.88	1.89	1.19
9	2-Octen-1-ol, (E)-	C_8_H_16_O	15.41	1041.72	1059	MS, RI	-	3.21	2.81	1.87	1.69	1.65	0.73	0.74	0.57	0.64	0.34
10	3,5-Octadien-2-ol	C_8_H_14_O	15.80	1053.31	1039	MS, RI	-	-	-	-	-	-	-	1.67	-	-	-
11	1-Octanol	C_8_H_18_O	16.88	1086.07	1087	MS, RI	-	78.97	95.89	-	-	-	-	-	-	-	-
12	5-Octen-2-ol, 5-methyl-	C_9_H_18_O	17.54	1106.30	-	MS	-	30.29	24.30	19.03	17.98	17.88	14.98	15.08	13.68	9.62	9.80
13	3-Decyn-2-ol	C_10_H_18_O	17.70	1111.55	1101	MS, RI	-	3.05	-	-	-	-	-	-	-	-	-
14	2-Nonen-1-ol, (E)-	C_9_H_18_O	19.91	1183.74	1171	MS, RI	-	-	-	-	3.97	3.22	1.82	1.34	1.13	1.23	1.50
15	1-Nonanol	C_9_H_20_O	19.99	1186.58	1186	MS, RI	-	13.28	23.21	22.71	45.23	25.13	17.42	14.07	12.11	12.34	13.38
16	1-Decanol	C_10_H_22_O	22.90	1288.14	1279	MS, RI	-	-	-	-	-	-	-	1.96	-	-	-
**Esters**																	
1	Formic acid, hexyl ester	C_7_H_14_O_2_	11.87	941.92	927	MS, RI	-	-	-	-	2.54	2.09	2.66	1.98	1.07	1.27	1.01
2	Formic acid, octyl ester	C_9_H_18_O_2_	16.90	1086.66	-	MS	-	-	87.96	77.06	132.20	68.07	62.10	55.59	43.15	39.37	32.29
3	1-Octen-3-yl-acetate	C_10_H_18_O_2_	17.25	1097.11	1097	MS, RI	-	-	-	-	-	1.20	1.09	1.34	1.32	1.02	1.21
4	Acetic acid, octyl ester	C_10_H_20_O_2_	20.34	1197.97	1200	MS, RI	-	-	-	-	7.83	3.29	2.44	2.09	1.79	1.23	1.24
5	Oxirane, decyl-	C_12_H_24_O	23.51	1310.47	1307	MS, RI	-	2.43	2.48	2.07	-	-	-	-	-	-	-
**Aromatic hydrocarbons**																	
1	Cyclopentane, methyl-	C_6_H_12_	2.65	626.43	624	MS, RI	-	1.86	1.65	-	-	-	-	-	-	-	-
2	*n*-Hexane	C_6_H_14_	3.46	687.93	-	MS	-	-	-	2.52	-	-	-	-	-	-	-
3	Heptane	C_7_H_16_	3.58	697.48	-	MS	-	-	-	-	8.38	5.40	2.30	1.83	1.99	2.28	2.21
4	1-Octene	C_8_H_16_	6.53	797.74	794	MS, RI	3.94	4.93	4.33	5.91	-	-	-	-	-	-	-
5	Hexane, 2,4-dimethyl-	C_8_H_18_	6.81	807.75	-	MS	-	-	-	-	-	15.65	7.43	7.78	1.62	-	-
6	1-Nonene	C_9_H_18_	9.70	883.77	893	MS, RI	46.10	15.82	11.12	5.48	-	-	-	-	-	-	-
7	Octane, 2,7-dimethyl-	C_10_H_22_	11.55	933.18	934	MS, RI	3.33	-	-	-	-	-	-	-	-	-	-
8	4,6-Decadiene	C_10_H_18_	18.53	1133.78	1145	MS, RI	4.02	3.96	4.23	4.29	4.33	3.02	2.78	1.40	1.40	-	-
9	4-Dodecyne	C_12_H_22_	20.45	1201.69	1203.00	MS, RI	-	-	-	1.76	-	-	-	-	-	-	-
10	3-Dodecyne	C_12_H_22_	20.78	1213.20	1210	MS, RI	3.37	-	-	-	-	-	-	-	-	-	-
11	1-Octadecyne	C_18_H_34_	21.56	1240.88	1238	MS, RI	3.12	3.13	-	-	-	-	-	-	-	-	-
12	5-Tridecene, (Z)-	C_13_H_26_	22.48	1273.35	1272.8	MS, RI	2.21	1.98	1.60	1.18	1.13	1.23	0.93	0.99	0.82	0.96	1.38
13	2-Dodecenal, (E)-	C_12_H_22_O	27.56	1457.11	1452	MS, RI	-	1.24	1.45	1.52	1.23	1.06	0.22	-	-	-	-
**Internal standard**																	
1	1,2-Dichloro-benzene	C_6_H_4_Cl_2_	14.89	1018	1014	MS, RI	10	10	10	10	10	10	10	10	10	10	10

^1^ RT, retention time; ^2^ RI, retention index. The RI of unknown compounds in an HP-5MS column calculated against the GC-MS retention time of *n*-alkanes (C3-C25); ^3^ RI from a database (http://webbook.nist.gov/chemistry); ^4^ RI, agreed with the retention index from the literature; MS, compared with NIST 11 Mass Spectral Database; STD, agreed with the mass spectrum of standard chemical; ‘-’, not detected; ^5^ F, fermentation; ^6^ S, storage.

**Table 2:** Odor activity values (OAVs) for compounds produced in milk fermented by *S. thermophilus* MGA45-4 during fermentation and storage.

Volatile Compound	Chemical Formula	Odor threshold (μg/L)	References	OAV										
				0 h (F) ^1^	2 h (F)	4 h (F)	6 h (F)	0 d (S) ^2^	12 h (S)	1 d (S)	2 d (S)	3 d (S)	7 d (S)	14 d (S)
Butanal, 3-methyl-	C_5_H_10_O	5.4	[42]	1.39	2.78	3.14	2.34	-	-	-	-	-	-	-
2-Pentenal, (E)-	C_5_H_8_O	1.2	[38]	-	-	-	1.61	0.96	0.86	0.85	1.03	1.04	1.33	1.57
Hexanal	C_6_H_12_O	3	[38]	33.03	30.53	19.37	17.34	1.32	-	-	-	-	-	-
2-Heptenal, (E)-	C_7_H_12_O	13	[43]	0.77	0.88	0.80	0.89	1.81	0.57	0.26	0.48	0.23	0.38	0.44
Octanal	C_8_H_16_O	0.7	[38]	127.93	64.46	33.56	26.06	11.04	4.21	-	-	-	-	-
2-Octenal, (E)-	C_8_H_14_O	3	[44]	4.43	4.62	5.02	5.22	6.83	3.40	1.58	1.48	1.19	1.16	1.43
Nonanal	C_9_H_18_O	1	[45]	52.04	35.95	28.60	9.58	4.01	1.52	1.43	1.03	1.50	1.12	1.13
2,3-Butanedione	C_4_H_6_O_2_	10	[42]	-	-	-	-	1.61	1.65	1.53	1.61	0.90	-	-
Acetoin	C_4_H_8_O_2_	55	[44]	-	-	0.06	0.37	1.06	0.50	0.54	0.44	0.38	0.44	0.44
Hexanol	C_6_H_14_O	120	[42]	0.34	0.36	0.41	0.45	1.27	0.62	0.37	0.32	0.36	0.36	0.34
1-Octen-3-ol	C_8_H_16_O	10	[23]	0.49	0.74	0.45	0.58	0.99	0.42	0.22	0.17	0.11	0.19	0.11

^1^ F, fermentation; ^2^ S, storage.
